# Hebra’s Management of Scabies and Eczema

**Published:** 1869-08

**Authors:** 


### Hebra's Management of Scabies and Eczema.

This distinguished professor of cutaneous diseases treats all his poor patients affected with *Scabies* with the application of his "Cure Salve." At his clinique a half-dozen men and boys, perfectly naked, are drawn up in line, and the old nurse takes his position before them, with a large pot of the semi-solid salve at his feet.

A small quantity is presented to each for the hands, then the forearms, arms, abdomen, lower extremities, &c., and finally for the back; the company then faces to the right, standing in company file, when each officiates for the man before. After the friction, each individual is laid in bed and rolled in a blanket from head to foot, and there he lies for two days. He is then inspected; should he not require another application, he is now rubbed off with fine clay. Prof. H. insists strongly that the patient shall not bathe for four or five days, as the concomitant eczema is always aggravated thereby. Should the eczema require treatment, the patient is furnished with ol. cadini, a preparation of tar, which he is instructed to use. The composition of the all-healing salve is as follows: Sulphur, tar, each six ounces; soap, fat, each one pound; chalk, four ounces.

*Eczema* is treated according to its form. In an acute case which occurred recently, wherein the entire trunk was covered with vesicles on an inflamed base, pulverized starch was applied, and in four or five days the cure was complete. Incrustations are softened and removed by the application of cloths saturated in cod-liver oil; when either the oil is continued, or preparations of tar united with it, or alone maintained, or in obstinate forms, a caoutchouc glove or stocking is worn.—*Vienna Correspondence of Western Journal of Medicine.—Medical Record.*

